# Artemisinin resistance – modelling the potential human and economic costs

**DOI:** 10.1186/1475-2875-13-452

**Published:** 2014-11-23

**Authors:** Yoel Lubell, Arjen Dondorp, Philippe J Guérin, Tom Drake, Sylvia Meek, Elizabeth Ashley, Nicholas PJ Day, Nicholas J White, Lisa J White

**Affiliations:** Mahidol-Oxford Tropical Medicine Research Unit (MORU), Faculty of Tropical Medicine, Mahidol University, Bangkok, Thailand; Centre for Tropical Medicine, Nuffield Department of Medicine, Oxford, UK; Worldwide Antimalarial Resistance Network (WWARN), Churchill Hospital, University of Oxford, Oxford, UK; The Malaria Consortium, London, UK

**Keywords:** Malaria, Artemisinins, Anti-malarial resistance, Economics

## Abstract

**Background:**

Artemisinin combination therapy is recommended as first-line treatment for falciparum malaria across the endemic world and is increasingly relied upon for treating vivax malaria where chloroquine is failing. Artemisinin resistance was first detected in western Cambodia in 2007, and is now confirmed in the Greater Mekong region, raising the spectre of a malaria resurgence that could undo a decade of progress in control, and threaten the feasibility of elimination. The magnitude of this threat has not been quantified.

**Methods:**

This analysis compares the health and economic consequences of two future scenarios occurring once artemisinin-based treatments are available with high coverage. In the first scenario, artemisinin combination therapy (ACT) is largely effective in the management of uncomplicated malaria and severe malaria is treated with artesunate, while in the second scenario ACT are failing at a rate of 30%, and treatment of severe malaria reverts to quinine. The model is applied to all malaria-endemic countries using their specific estimates for malaria incidence, transmission intensity and GDP. The model describes the direct medical costs for repeated diagnosis and retreatment of clinical failures as well as admission costs for severe malaria. For productivity losses, the conservative friction costing method is used, which assumes a limited economic impact for individuals that are no longer economically active until they are replaced from the unemployment pool.

**Results:**

Using conservative assumptions and parameter estimates, the model projects an excess of 116,000 deaths annually in the scenario of widespread artemisinin resistance. The predicted medical costs for retreatment of clinical failures and for management of severe malaria exceed US$32 million per year. Productivity losses resulting from excess morbidity and mortality were estimated at US$385 million for each year during which failing ACT remained in use as first-line treatment.

**Conclusions:**

These ‘ballpark’ figures for the magnitude of the health and economic threat posed by artemisinin resistance add weight to the call for urgent action to detect the emergence of resistance as early as possible and contain its spread from known locations in the Mekong region to elsewhere in the endemic world.

## Background

The past decade has seen substantial progress in malaria control, with local elimination now a feasible objective in parts of the Asia-Pacific, Middle East, Latin America and even in some areas of sub-Saharan Africa (SSA). Much of this progress has been ascribed to the increasing availability of artemisinin compounds, with their rapid clearance of asexual blood stage parasitaemia and gametocytocidal properties that curb transmission to other individuals [1, 2]. Artemisinin-based combination therapy (ACT) is the recommended first-line treatment for uncomplicated falciparum malaria across almost the entire endemic world, and large investments are being made to extend coverage in both the public and private sectors. For severe malaria, the recent change in recommended treatment from quinine to artesunate offers an approximately 25% greater chance of survival [3, 4] although uptake in endemic areas has been slow.

The loss of artemisinin efficacy would therefore threaten these real and potential gains, and historical precedent, clinical, laboratory, and modelling work all suggest that artemisinin compounds could lose their efficacy long before elimination is a realistic aim in high transmission areas [4–9].

Evidence to suggest emergence and/or spread of artemisinin resistance is amassing. Early warning signs in the form of slowing parasite clearance times in western Cambodia were detected in 2007 and soon after along the Thai-Burmese border [5, 10, 11]. There is now evidence of artemisinin resistance in *Plasmodium falciparum* in five countries in the Greater Mekong region [12]. The recent identification of Kelch mutations associated with artemisinin resistance on chromosome 13 is likely to better specify just how far it has already spread [13].

In ACT, the loss of artemisinin efficacy would expose partner drugs to greater selection pressure for the development of resistance, compromising the effectiveness of the combination. There is currently no good alternative to ACT suitable for large-scale implementation. New drugs will surely be developed but the lag time between development, registration, change of national treatment policy, training, and large-scale production imply an inevitable and costly delay until affordable substitutes to ACT are widely available. For severe malaria the spread of resistance will likely result in reverting to or maintaining quinine as the treatment of choice, and therefore the loss of the real or potential gain offered by artesunate.

The following analysis is a modelled snapshot of two contrasting future scenarios following extensive adoption of ACT. In the first, artemisinins maintain high levels of efficacy with ACT cure rates of 95% and where artesunate is used to treat severe malaria. In the second scenario, artemisinins face widespread resistance, leading to ACT clinical failure rates of 30% and where policy has reverted to quinine to manage severe malaria. This is not a prediction of how artemisinin resistance is likely to spread and result in clinical failure of ACT, or the interaction with changing malaria transmission. Rather, the aim here is to estimate the magnitude of the threat posed by artemisinin resistance should this result in increasing ACT failures and the loss of artesunate’s advantage in treating severe malaria.

## Methods

The excess mortality associated with artemisinin resistance is a product of: i) an increased proportion of ACT failures in uncomplicated malaria, a proportion of which become severe; and, ii) patients with severe malaria who are treated with quinine instead of artesunate [4]. The economic costs comprise of additional diagnostic tests and ACT for treatment failures, the cost of treating a higher number of severe malaria cases, and the cost of switching policy to alternative ACT or other first-line therapy once these are available. Productivity losses associated with the excess morbidity and mortality are also estimated.

### Baseline and resistance scenarios

This analysis compares a baseline scenario characterized by an ACT failure rate of 5% against a scenario of 30% ACT failure rate, a conservative estimate compared with the fate of previous first-line treatments [14] and with a recent study from the northwestern border of Thailand, an area where artemisinin resistance is established, where the failure rate for artesunate-mefloquine in the treatment of *Plasmodium falciparum* was 58% [10]. Severe malaria cases that access inpatient care are assumed to receive artesunate in the baseline scenario and quinine in the scenario of artemisinin resistance. The excess impact is calculated by subtracting the total mortality and costs of the baseline scenario from those of the resistance scenario. ACT coverage is assumed to be complete in both future scenarios, as is artesunate coverage for severe malaria in the baseline scenario.

### Model structure and parameter estimates

**Figure 1:**
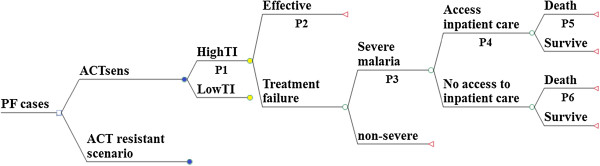
**Decision tree model of the human and economic consequences of artemisinin-combination therapy malaria treatment failure.** The decision tree diagram illustrates how malaria incidence, deaths and costs are calculated in each of the two scenarios. The top branch represents the scenarios in which artemisinins are effective. This structure is replicated in the bottom branch representing the scenario of artemisinin resistance, with the necessary adjustments to parameter values. The branch following the blue node at *High transmission* is also replicated with parameter adjustments at the *Low transmission* node.

A decision tree was used to model the outcomes of the two scenarios (Figure 1). The model is applied to each malaria-endemic country with outputs being aggregated by region. The WHO estimates for country-level incidence of presumed and confirmed malaria cases from the 2013 World Malaria Report were used as these are conservative estimates that include cases that are most likely to receive an ACT, as opposed to other estimates that include undocumented cases, which by definition are seeking care outside of the formal health sector. The number of cases was modified by the proportion of falciparum cases from the same report. The model then divides patients between low and high transmission settings, using country-specific data, with implications for population immunity and subsequent mortality rates.

The model subdivides the cases into treatments that were effective and those that fail; in the baseline scenario a failure rate of 5% is assumed [14] while in the resistance scenario a conservative estimate of 30% is used. A small proportion of treatment failures deteriorate to severe illness with a point estimate of 2%. This is derived from an artesunate efficacy study for uncomplicated malaria in areas of Cambodia where resistance is believed to be high, where 3/159 patients developed severe illness [15]. A proportion of severe cases are able to access inpatient care; in the absence of relevant data, a range of estimates from 40 to 90% were used, determined by a log function of the countries’ GDP per capita. For patients without access to care, a high mortality rate of 50 and 70% was assumed in high- and low-transmission settings [16]. For patients who access health facilities, an assumption is made that in the baseline scenario first-line treatment is artesunate with a mortality rate of 8.5 and 15% in high- and low-transmission settings, respectively. In the scenario of artemisinin resistance, it is assumed that severe malaria would be treated with quinine [14], associated with a mortality of 10.9% in high-transmission areas and 22% in other regions [3, 4].

### Direct medical costs, policy-changing costs and productivity losses

The excess cost of a treatment failure that does not progress to severe illness was estimated as the cost of an additional diagnostic test and a second ACT (again, assuming full coverage). A lower unit cost per ACT of 0.8 US$ in applied to the high-transmission setting where most patients will be young children and 1.6 US$ per ACT in the low-transmission setting where most patients are adults, and a cost of 0.8 US$ per rapid diagnostic test [17]. The cost of inpatient care for severe malaria was estimated at 65 US$ [18].

The introduction of a new first-line therapy, once available, will require policy changes with supporting and training programmes to facilitate its deployment. Mulligan *et al.* conducted the only detailed estimate for the costs of a national revision of anti-malarial treatment guidelines, for Tanzania in 2002 [19]. These costs include retraining and education programmes but not the incremental costs of newer drugs, which by historical precedent would exceed those of their predecessors. After inflationary adjustments and classification as either fixed or variable costs, the latter assumed to be correlated with population size, these findings were applied to other malaria-endemic countries.

Productivity losses are estimated using GDP per capita for each country [20]. Productivity losses due to excess morbidity are calculated using an estimate of one week of lost earnings due to a case of uncomplicated malaria [21], an additional week of productivity lost to treatment failure, and an assumed three weeks of lost productivity due to severe malaria. Productivity losses for mortality are equated to those of severe illness, adopting the rationale of the friction cost methodology that assumes only a short-term impact on productivity, with an unemployment reservoir mitigating a longer term economic impact [22]. This is a conservative methodology as compared with the more widely used human capital approach that equates productivity losses with life-long lost earnings, as this has been criticised for inflating the actual economic impact [23].

Costs are expressed in 2013 USD. Where adjustments were necessary we used the World Bank consumer price index and exchange rates to adjust costs from local units to 2013 USD.

### Sensitivity analysis

A probabilistic sensitivity analysis was carried out to generate results using distributions for the probability of treatment failures becoming severely ill and for the mortality rates for treated and untreated severe malaria (shown in Table 1), with 1,000 random samples drawn from each distribution and for each country. The mean mortality and costs for each scenario were used to obtain the point estimates and the values at the 2.5 and 97.5% quintiles for the uncertainty intervals.

**Table 1:** **Parameter estimates, ranges and sources**

Parameter	Base case	Range/distribution	Comments	Source
**Probabilities for clinical parameters**				
ACT failure rate in a scenario of widespread resistance (P2)	30%	30-80%	A conservative estimate as compared with recent ACT failure rates in Mae Sot, Thailand and those for chloroquine, amodiaquine and sulphadoxine-pyrimethamine in SSA and Asia.	Assumption [10, 14]
ACT failure rates in the absence of widespread resistance (P2)	5%	0-10%		[14]
Treatment failure becomes severe (P3)	2%	0.5-5%	Data from an artesunate efficacy trial in Cambodia and best fit to WHO incidence/mortality data	[15, 24]
		Beta distribution		
		(α = 3 β = 156)		
Mortality rate for severe malaria treated with quinine – Asia, EM, LA	22%	Beta distribution	A large multisite in Asia (the trial data were also used to construct the probability distributions for the PSA)	[3]
		(α = 164 β = 567)		
Mortality rate for severe malaria treated with artesunate – Asia, EM, LA (P5)	15%	Beta distribution		[3]
		(α = 107 β = 627)		
Mortality rate for severe malaria treated with quinine – SSA (P5)	10.9%	Beta distribution	The largest study of severe malaria treatments in hospitalized patients	[4]
		(α = 297 β = 2,416)		
Mortality rate for severe malaria treated with artesunate – SSA (P5)	8.5%	Beta distribution		[4]
		(α = 230 β = 2,482)		
Mortality rate for untreated severe malaria – high transmission (P6)	50%	40-90%		[16, 24]
		Beta distribution		
		(α = 5 β = 5)		
Mortality rate for untreated severe malaria – low transmission (P6)	75%	40-90%		[16, 24]
		Beta distribution		
		(α = 7 β = 3)		
**Access to care**				
Access to any anti-malarial (P1)		20-100%	Country level data	[24–27]
Access to inpatient care (P4)		40-90%	Determined by GDP per capita	
**Costs**				
ACT	$0.8/$1.6		Private sector prices are mostly higher which would imply higher costs for retreatment of failures	[28]
Test	$0.8	$0.5-1.5		[24]
Inpatient care for severe malaria	$65			[18]

SSA – sub-Saharan Africa; LA – Latin America; EM – Eastern Mediterranean; SEA – Southeast Asia; WP – Western Pacific.

The sensitivity of results to individual parameters was tested by varying their values within plausible ranges (shown in Table 1) while holding other parameters at their initial estimates. The impact of the most influential parameters were summarized in a Tornado diagram and in two-way sensitivity analyses. As model parameters are specific to each region or country, the sensitivity analyses focussed on SSA where the burden of malaria and the impact of artemisinin resistance would be highest.

The primary analysis assumes full ACT coverage. Lower coverage of ACT would imply a lesser potential impact of widespread resistance (but lesser gains due to ACT in the baseline scenario in the first place). The other assumption used in constructing the scenarios was a clinical failure rate in ACT of 30% in the presence of widespread artemisinin resistance. The impact of using different assumptions in defining the scenario of artemisinin resistance was explored, assuming that in the absence of an ACT the coverage of anti-malarials is similar to current best estimates (using the most recent country specific data from DHS surveys [29]) and with an efficacy in non-artemisinin-based anti-malarials of 35%, a higher estimate than the data for most non-artemisinin anti-malarials would suggest [13].

The model was developed using TreeAge Pro (TreeAge Software Inc, Williamstown, MA, USA) and results were graphed using Microsoft Excel and mapped in Google Maps.

## Results

**Figure 2:**
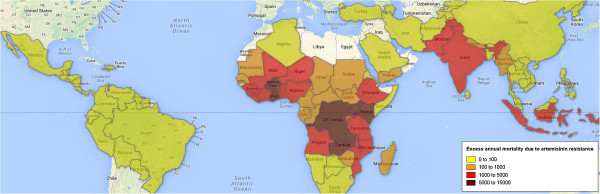
**Excess mortality due to artemisinin and artemisinin-combination therapy resistance in malaria-endemic areas.** The map shows the model output for estimated excess mortality in the scenario of artemisinin resistance. Individual country estimates for this and other model outputs are available online [30].

**Figure 3:**
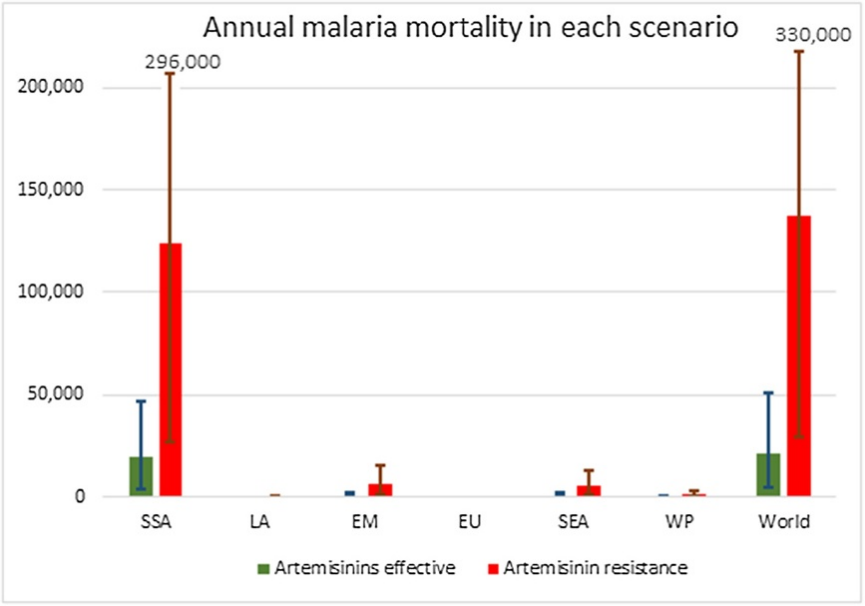
**Annual malaria mortality in each of the two scenarios.** Malaria mortality in each of the two scenarios by region. SSA – sub-Saharan Africa; LA – Latin America; EM – Eastern Mediterranean; SEA – Southeast Asia; WP – Western Pacific.

If malaria incidence was to remain similar to current levels, the model estimates the excess number of treatment failures in the scenario of widespread artemisinin resistance to approximate 22 million annually, until an effective alternative anti-malarial is deployed. These would lead to 230,000 additional severe malaria cases (surviving) and 116,000 excess deaths per year. Excess malaria mortality estimates by country are presented in Figure 2 and the annual mortality in each scenario by region in Figure 3, as well as the 95% uncertainty interval for these from the probabilistic sensitivity analysis.

Approximately 77% of these deaths would result from the excess number of ACT treatment failures in the scenario of artemisinin resistance, while the remaining 23% are due to the use of quinine instead of artesunate for the management of severe malaria.

The direct medical costs for malaria treatments in the baseline scenario would be 114 million US$ (111–117 million US$), while in the scenario of ACT resistance this would be 28% higher at 146 million US$ (134–167 million US$). The cost of policy change across the endemic world is estimated at 130 million US$. This cost would be borne repeatedly if switching between ACT as and when resistance to the partner drug emerged, and once an alternative non-artemisinin-based class of drug is deployable.

**Figure 4:**
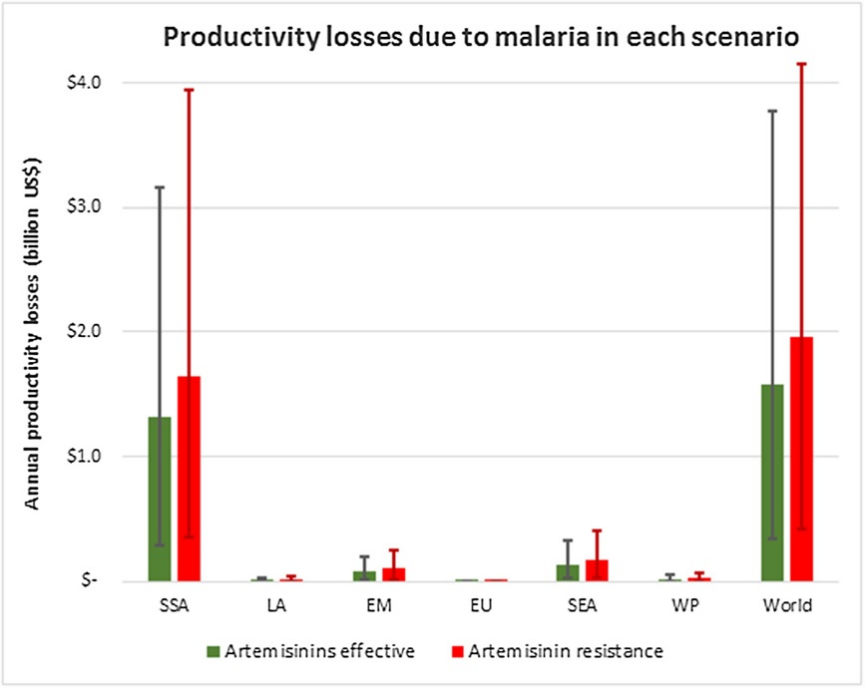
**Productivity losses due to artemisinin resistance.** These values represent the productivity losses each year in the scenario of widespread resistance using the conservative friction cost method.

The model estimates productivity losses due to excess morbidity and mortality at 385 million US$ when using the conservative friction cost methodology (regional break down shown in Figure 4). Approximately 90% of this cost is due to productivity losses associated with excess morbidity following treatment failures. Country-specific model outputs for mortality and costs in each of the two scenarios are available on the interactive map online [30].

### Sensitivity analysis

**Figure 5:**
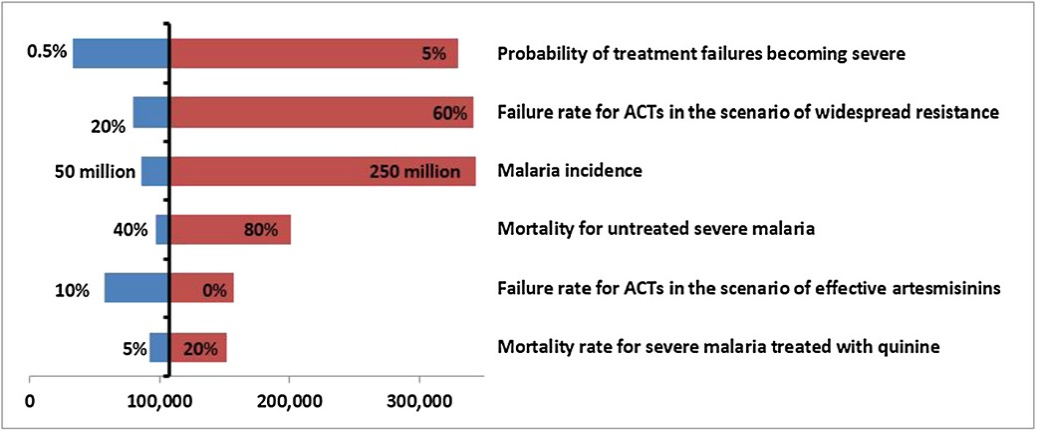
**The sensitivity of projected excess deaths in sub-Saharan Africa to key input parameters.** The graph illustrates the relative impact of different parameters on model outputs. A key parameter is the probability of patients with a treatment failure becoming severely ill. A higher estimate of 5% implies a large increase in the total excess mortality in SSA to over 300,000 deaths per year. Another influential parameter is the treatment failure rate for ACT in the scenario of widespread resistance. If clinical failure rates were to resemble those documented in many previously used antimalarials the excess mortality would be far higher.

A large number of parameters were influential in determining the excess mortality, most importantly the probability of treatment failures becoming severe, the incidence of malaria cases, and the mortality rate in severe malaria treated with quinine (Figure 5). The probability of treatment failures becoming severely ill was particularly influential with a range of 0.5 to 5% corresponding to excess deaths ranging from 25,000 to over 300,000. Similar influences are observed in the excess costs as these are a product of the excess clinical failures, severe cases and deaths.

**Figure 6:**
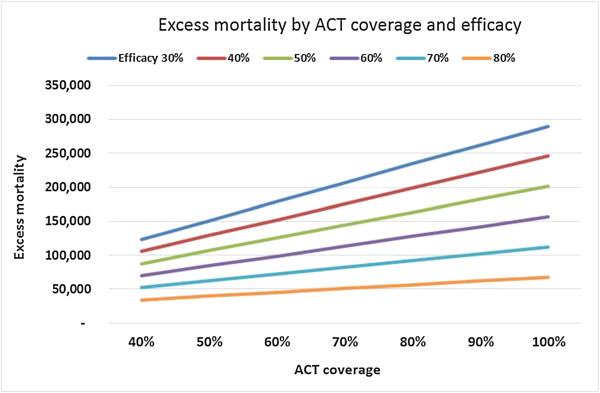
**Excess mortality in sub-Saharan Africa in the scenario of artemisinin resistance across varying levels of artemisinin-combination therapy coverage and efficacy rates for artemisinin-combination therapy in the resistance scenario.** Lower coverage of ACT would imply a lesser impact in the scenario of artemisinin resistance (and conversely lower potential benefit in the scenario of effective artemisinins). Varying the degree of ACT efficacy in the scenario of artemisinin resistance would have a large impact on results.

Figure 6 shows the impact of varying the ACT coverage and their effectiveness in the scenario of artemisinin resistance. With the lowest estimate for efficacy of 30% (still exceeding that for widely used non-artemisinin treatments in many endemic areas), the mortality estimate for SSA rises to approximately 300,000. If, however, the coverage of ACT remains restricted to 40% of cases, the impact of artemisinin resistance will range between approximately 35,000 and 130,000 excess deaths, depending on the estimate for failure rates.

## Discussion

This analysis does not project how artemisinin resistance is likely to spread or portray the most probable scenario that is likely to unfold. Rather, this is a crude assessment of the threat posed by artemisinin resistance in the event that efficacy drops to 70%, which would remain more efficacious than most previous first-line anti-malarials once resistance to these emerged. Many key parameter estimates are uncertain, although the methods and point estimates chosen were conservative. The scope of cost elements and economic impacts included was also limited. Household costs due to malaria were excluded although these have been shown to be substantial [31] and are also likely to increase with ineffective treatments. Artemisinin resistance is likely to threaten elimination strategies, allowing malaria to continue its demonstrated drain on macro-economic growth [32, 33]. Re-introduction of malaria to areas that have recently eliminated is also more likely with loss of effective treatment and costly surveillance systems will be required as long as this remains a possibility. Inclusion of these factors would imply greater health and economic costs than those described above.

The analysis considered homogenous ACT failure rates across the endemic world, but which areas are most likely to be affected? While the projected burden in this analysis is greatest in Africa, there is no evidence to date that resistance has spread there. Countries in closer proximity to the known epicentre, such as Myanmar, Bangladesh and India with large populations at risk of unstable *P falciparum* transmission, face a more imminent threat. Asian areas of low transmission with high drug pressure continue to provide fertile breeding ground for the emergence and spread of drug resistance. Use of artemisinin monotherapy was widespread in much of the region and substandard artemisinin-based treatments are also prevalent [34, 35]. For these reasons resistance to all previous anti-malarials was documented initially in Southeast Asia, which was followed by its spread to Africa where the impact was most detrimental. Greater population movement could further facilitate the spread of artemisinin resistance as compared with previous anti-malarials. Increasing migration between China and Africa, for instance, could facilitate transmission of resistant parasites between these regions [36].

There are extensive limitations to this analysis relating to both the model structure and parameter estimates. The model’s static structure compares two distinct scenarios – ACT being effective to a homogenous 30% failure rate for all ACT across the endemic world. In reality the spread and impact of artemisinin resistance followed by ACT resistance, should it occur, will be extremely heterogeneous and dependent on transmission intensity, coverage, partner drug mutation rates, health system preparedness, and many other variables. Containment measures could mitigate the spread of resistance. It might be possible, for instance, to ‘buy time’ by extending the regimen of ACTs to maintain high cure rates. Alternatively other ACT with new partner drugs could be introduced if existing ones fail, mitigating some of the potential impact but incurring the cost of policy change and likely higher drug costs. Exclusion of these factors from the analysis is an over-simplification but is nevertheless consistent with the aim of quantifying the cost of widespread artemisinin and ACT resistance should this occur. Some key parameter estimates are lacking robust supporting evidence, most importantly the probability of becoming severely ill following treatment failure for which we used a single study with patients treated with artesunate.

Critically, the model assumes that the incidence of malaria in both scenarios will resemble current levels. For the annual incidence, the number of probable and confirmed malaria cases in the 2013 World Malaria Report was used. These are at the lower end of current estimates [24, 37] and do not account for many undocumented cases, but this is more consistent with the assumption of high future ACT coverage, as these cases will have been appropriately diagnosed, treated and documented. In addition to the extensive uncertainty surrounding incidence for previous years, projections of future trends are challenging, with transmission being influenced by the availability of ACT and other interventions, as well as a range of factors including economic, environmental and demographic change. The spread of artemisinin and ACT resistance will itself be a product as well as a cause of change in transmission. A global malaria transmission model accounting for all these factors required to predict the spread of artemisinin resistance has not yet been successfully created.

## Conclusion

This analysis, albeit dependent on many strong assumptions, provides a set of conservatively estimated ‘ballpark’ figures for the excess mortality and economic losses that would follow widespread resistance to artemisinin-based therapy. These figures in themselves cannot be used to identify the optimal levels of investment that would be justified to contain artemisinin resistance, as this would require further estimates for the probability that a scenario such as this will unfold and the potential effectiveness of containment strategies. The magnitude of the threat, however, suggests that even if this is a remote possibility, considerably greater attention and investment in delaying or eliminating its possible emergence than are currently being provided are justified. There may be only a limited window of opportunity to contain and eliminate this imminent global threat.

### Ethics

No specific ethical approval was sought or deemed necessary for this study.

## References

[1] O’Meara WP, Mangeni JN, Steketee R, Greenwood B (2010). Changes in the burden of malaria in sub-Saharan Africa. Lancet Infect Dis.

[2] Sawa P, Shekalaghe SA, Drakeley CJ, Sutherland CJ, Mweresa CK, Baidjoe AY, Manjurano A, Kavishe RA, Beshir KB, Yussuf RU, Omar SA, Hermsen CC, Okell L, Schallig HD, Sauerwein RW, Hallett RL, Bousema T (2013). Malaria transmission after artemether-lumefantrine and dihydroartemisinin-piperaquine: a randomized trial. J Infect Dis.

[3] Dondorp A, Nosten F, Stepniewska K, Day N, White N (2005). Artesunate versus quinine for treatment of severe falciparum malaria: a randomised trial. Lancet.

[4] Imwong M, Dondorp AM, Nosten F, Yi P, Mungthin M, Hanchana S, Das D, Phyo AP, Lwin KM, Pukrittayakamee S, Lee SJ, Saisung S, Koecharoen K, Nguon C, Day NP, Socheat D, White NJ (2010). Exploring the contribution of candidate genes to artemisinin resistance in *Plasmodium falciparum*. Antimicrob Agents Chemother.

[5] Dondorp AM, Nosten F, Yi P, Das D, Phyo AP, Tarning J, Lwin KM, Ariey F, Hanpithakpong W, Lee SJ, Ringwald P, Silamut K, Imwong M, Chotivanich K, Lim P, Herdman T, An SS, Yeung S, Singhasivanon P, Day NP, Lindegardh N, Socheat D, White NJ (2009). Artemisinin resistance in *Plasmodium falciparum* malaria. N Engl J Med.

[6] Saralamba S, Pan-Ngum W, Maude RJ, Lee SJ, Tarning J, Lindegardh N, Chotivanich K, Nosten F, Day NP, Socheat D, White NJ, Dondorp AM, White LJ (2011). Intrahost modeling of artemisinin resistance in *Plasmodium falciparum*. Proc Natl Acad Sci U S A.

[7] Pongtavornpinyo W, Hastings IM, Dondorp A, White LJ, Maude RJ, Saralamba S, Day NP, White NJ, Boni MF (2009). Probability of emergence of antimalarial resistance in different stages of the parasite life cycle. Evol Appl.

[8] Maude RJ, Pontavornpinyo W, Saralamba S, Aguas R, Yeung S, Dondorp AM, Day NP, White NJ, White LJ (2009). The last man standing is the most resistant: eliminating artemisinin-resistant malaria in Cambodia. Malar J.

[9] White LJ, Lubell Y, Meek S, White NJ, Day NP, Nosten F, Ashley E, Socheat D, Nguon C, Dondorp A (2012). Malaria in the Asia-Pacific: modelling the current and potential impact of artemisinin resistance and its containment. Malaria 2012 Saving Lives in the Asia Pacific.

[10] Carrara VI, Lwin KM, Phyo AP, Ashley E, Wiladphaingern J, Sriprawat K, Rijken M, Boel M, McGready R, Proux S, Chu C, Singhasivanon P, White N, Nosten F (2013). Malaria burden and artemisinin resistance in the mobile and migrant population on the thai-myanmar border, 1999–2011: an observational study. PLoS Med.

[11] Phyo AP, Nkhoma S, Stepniewska K, Ashley EA, Nair S, McGready R, Ler Moo C, Al-Saai S, Dondorp AM, Lwin KM, Singhasivanon P, Day NP, White NJ, Anderson TJ, Nosten F (2012). Emergence of artemisinin-resistant malaria on the western border of Thailand: a longitudinal study. Lancet.

[12] **Q&A on artemisinin resistance**. [http://www.who.int/malaria/media/artemisinin_resistance_qa/en/]

[13] Ariey F, Witkowski B, Amaratunga C, Beghain J, Langlois AC, Khim N, Kim S, Duru V, Bouchier C, Ma L, Lim P, Leang R, Duong S, Sreng S, Suon S, Chuor CM, Bout DM, Menard S, Rogers WO, Genton B, Fandeur T, Miotto O, Ringwald P, Le Bras J, Berry A, Barale JC, Fairhurst RM, Benoit-Vical F, Mercereau-Puijalon O, Menard D (2014). A molecular marker of artemisinin-resistant *Plasmodium falciparum* malaria. Nature.

[14] WHO (2010). Global Report on Antimalarial Drug Efficacy and Drug Resistance: 2000–2010.

[15] Das D, Tripura R, Phyo AP, Lwin KM, Tarning J, Lee SJ, Hanpithakpong W, Stepniewska K, Menard D, Ringwald P, Silamut K, Imwong M, Chotivanich K, Yi P, Day NP, Lindegardh N, Socheat D, Nguon C, White NJ, Nosten F, Dondorp AM (2013). Effect of high-dose or split-dose artesunate on parasite clearance in artemisinin-resistant falciparum malaria. Clin Infect Dis.

[16] Lubell Y, Staedke SG, Greenwood B, Kamya MR, Molineaux M, Newton PN, Reyburn H, Snow R, D’Alessandro U, English M, Day N, Kremsner P, Dondorp A, Mbacham W, Dorsey G, Owusu-Agyei S, Maitland K, Krishna S, Newton CR, Pasvol G, Taylor T, von Seidlein L, White NJ, Binka F, Mills A, Whitty CJ (2011). Likely health outcomes for untreated acute febrile illness in the tropics in decision and economic models; a Delphi survey. PLoS One.

[17] Pfeil J, Borrmann S, Tozan Y (2014). Dihydroartemisinin-piperaquine vs. artemether-lumefantrine for first-line treatment of uncomplicated malaria in African children: a cost-effectiveness analysis. PLoS One.

[18] Lubell Y, Riewpaiboon A, Dondorp AM, von Seidlein L, Mokuolu OA, Nansumba M, Gesase S, Kent A, Mtove G, Olaosebikan R, Ngum WP, Fanello CI, Hendriksen I, Day NP, White NJ, Yeung S (2011). Cost-effectiveness of parenteral artesunate for treating children with severe malaria in sub-Saharan Africa. Bull World Health Organ.

[19] Mulligan JA, Mandike R, Palmer N, Williams H, Abdulla S, Bloland P, Mills A (2006). The costs of changing national policy: lessons from malaria treatment policy guidelines in Tanzania. Trop Med Int Health.

[20] **GDP per capita (current US$)**. [http://data.worldbank.org/indicator/NY.GDP.PCAP.CD]

[21] Chuma J, Okungu V, Molyneux C (2010). The economic costs of malaria in four Kenyan districts: do household costs differ by disease endemicity?. Malar J.

[22] Koopmanschap MA, Rutten FF, van Ineveld BM, van Roijen L (1995). The friction cost method for measuring indirect costs of disease. J Health Econ.

[23] van den Hout WB (2010). The value of productivity: human-capital versus friction-cost method. Ann Rheum Dis.

[24] WHO (2012). World Malaria Report 2012.

[25] Littrell M, Gatakaa H, Evance I, Poyer S, Njogu J, Solomon T, Munroe E, Chapman S, Goodman C, Hanson K, Zinsou C, Akulayi L, Raharinjatovo J, Arogundade E, Buyungo P, Mpasela F, Adjibabi CB, Agbango JA, Ramarosandratana BF, Coker B, Rubahika D, Hamainza B, Shewchuk T, Chavasse D, O’Connell KA (2011). Monitoring fever treatment behaviour and equitable access to effective medicines in the context of initiatives to improve ACT access: baseline results and implications for programming in six African countries. Malar J.

[26] Littrell M, Gatakaa H, Phok S, Allen H, Yeung S, Chuor CM, Dysoley L, Socheat D, Spiers A, White C, Shewchuk T, Chavasse D, O’Connell KA (2011). Case management of malaria fever in Cambodia: results from national anti-malarial outlet and household surveys. Malar J.

[27] Tougher S, Ye Y, Amuasi JH, Kourgueni IA, Thomson R, Goodman C, Mann AG, Ren R, Willey BA, Adegoke CA, Amin A, Ansong D, Bruxvoort K, Diallo DA, Diap G, Festo C, Johanes B, Juma E, Kalolella A, Malam O, Mberu B, Ndiaye S, Nguah SB, Seydou M, Taylor M, Rueda ST, Wamukoya M, Arnold F, Hanson K (2012). Effect of the Affordable Medicines Facility-malaria (AMFm) on the availability, price, and market share of quality-assured artemisinin-based combination therapies in seven countries: a before-and-after analysis of outlet survey data. Lancet.

[28] O’Connell KA, Gatakaa H, Poyer S, Njogu J, Evance I, Munroe E, Solomon T, Goodman C, Hanson K, Zinsou C, Akulayi L, Raharinjatovo J, Arogundade E, Buyungo P, Mpasela F, Adjibabi CB, Agbango JA, Ramarosandratana BF, Coker B, Rubahika D, Hamainza B, Chapman S, Shewchuk T, Chavasse D (2011). Got ACTs? Availability, price, market share and provider knowledge of anti-malarial medicines in public and private sector outlets in six malaria-endemic countries. Malar J.

[29] **The DHS Program: demographic and health surveys**. [http://dhsprogram.com/Data/

[30] **Mapping the cost of widespread artemisinin resistance**. [http://goo.gl/oTg8rR]

[31] Chima RI, Goodman CA, Mills A (2003). The economic impact of malaria in Africa: a critical review of the evidence. Health Policy.

[32] Gallup JL, Sachs JD (2001). The economic burden of malaria. Am J Trop Med Hyg.

[33] Malaney P, Spielman A, Sachs J (2004). The malaria gap. Am J Trop Med Hyg.

[34] Nayyar GM, Breman JG, Newton PN, Herrington J (2012). Poor-quality antimalarial drugs in southeast Asia and sub-Saharan Africa. Lancet Infect Di.

[35] Tabernero P, Fernandez FM, Green M, Guerin PJ, Newton PN (2014). Mind the gaps–the epidemiology of poor-quality anti-malarials in the malarious world–analysis of the WorldWide Antimalarial Resistance Network database. Malar J.

[36] MacPherson DW, Gushulak BD, Baine WB, Bala S, Gubbins PO, Holtom P, Segarra-Newnham M (2009). Population mobility, globalization, and antimicrobial drug resistance. Emerg Infect Dis.

[37] Murray CJ, Rosenfeld LC, Lim SS, Andrews KG, Foreman KJ, Haring D, Fullman N, Naghavi M, Lozano R, Lopez AD (2012). Global malaria mortality between 1980 and 2010: a systematic analysis. Lancet.

